# Spatial and temporal changes in moth assemblages along an altitudinal gradient, Jeju-do island

**DOI:** 10.1038/s41598-022-24600-z

**Published:** 2022-11-29

**Authors:** Sei-Woong Choi, Jeong-Seop An, Jae-Young Lee, Kyung Ah Koo

**Affiliations:** 1 Department of Environmental Education, Mokpo National University, Muan, Jeonnam 58554 South Korea; 2 National Institute of Ecology, Seocheon, Chungnam 33657 South Korea; 3 Division for Natural Environment, Water and Land Research Group, Korea Environment Institute, Sejong, 30147 South Korea

**Keywords:** Ecology, Zoology

## Abstract

Montane species on islands attract attention due to their small and isolated populations and limited dispersal potential, making them vulnerable to extinction. We investigated the diversity pattern of moth assemblages over the 12-years-period (2009–2020) at 11 study plots on an island mountain (Mount Hallasan, Jeju-do Island, South Korea) to assess the changes in the moth assemblages in terms of species composition, richness, and abundances. We expected to find a decline in the number of species at these sites, given the reported decline in similar taxa in other temperate regions, such as Europe and North America. In contrast, we found that the numbers of species and individuals of moth populations on the island mountain have not significantly changed, except at the high-elevation sites, where the number of species has increased. Our results also show that the numbers of species and individuals are closely related to energy availability, actual evapotranspiration. Moreover, we found that the species composition during the study period has not been greatly changed, except at the lowermost and uppermost elevations. The mechanism driving this high dissimilarity of moth assemblages differed: the low-elevation site experienced high temporal turnover, and the high elevation sites also experienced high temporal turnover and nestedness resulting from active species replacement due to a recent forest fire and vegetation changes and the geographic and ecological constraints of the high elevations. To date, the moth species diversity of the temperate forests of the island mountain is not showing a drastic change. However, we observed that the moth assemblages had changed the number of species and individuals at low and high elevations. Given the biological and ecological limitations of moths (ectothermic organisms with limited habitat range) and considering the results of our study, we infer that climate change has impacted the diversity and species composition of moths on the island mountain.

## Introduction

Islands, which can be categorized as oceanic and continental shelves in terms of their origin, are an ideal natural laboratory for ecological and evolutionary research. Oceanic islands emerged from the sea as isolated landmasses, usually by volcanic activity. In contrast, continental shelf islands are formed by the isolation of a piece of the landmass formerly part of the larger mainland^1^. Continental islands include the continental shelf islands and volcanic islands, which have contact with the continent during the last glacial maximum and this contact allowed continental species to migrate and homogenize the species pool of most of these islands^2^. After the formation of an island or separation from the mainland, the species composition and diversity of these landmasses change in response to several factors, namely, geography (area, latitude, elevation, isolation, and geology), ecology (biotope availability and land use and management), biology (mobility, colonization capability, and presence of organisms), and time^1,3–6^.

The cone-shaped geometry of islands demonstrates a different level of elevational gradients but the elevational range was significantly lower on islands than on the mainland and shows less elevational heterogeneity due to the limited area of an intrinsic feature of the island^2^. It has often been argued that the area effect on species richness was more apparent on islands than on mainlands^7,8^. However, the studies showed that the species-area relationship was not different between islands and mainlands^9^, and the main driving forces of species richness such as climate and area worked in the same way in both islands and mainlands^10^.

The diversity pattern along elevational gradients in subtropical and tropical mountains is well understood^11–15^. Drivers for the diversity pattern along elevational gradients are variable depending on the taxa and the study area. Climate-related variables such as temperature, precipitation, and net primary productivity are found to be strong predictors of species richness along elevational gradients^16–19^. In addition, spatial variables such as area and habitat heterogeneity could be important drivers of elevational richness patterns^18–20^. Islands are significantly cooler, wetter, and less seasonal than mainland^2^. However, it was noted that the main components of ecological structure and function such as climate, habitat, topography, soil, and time played equally on islands and terrestrial ecosystem^21^.

Montane species on islands are often endemic to a single mountain range^22^. Such species, which tend to be distributed in small populations isolated from source populations, are limited by the elevation range of the island and are at risk of population decline due to environmental changes, including climate change^23^. On Mount Hallasan National Park (HNP), approximately 22 endemic plant species and one endemic butterfly species, *Eumenis autonoe* (Esper) are found at elevations greater than 1500 m a.s.l. and the viability of these populations on the mountain is severely threatened by climate change^24^.

Moths are a megadiverse insect group, comprising more than 160,000 species that play essential roles as herbivores and pollinators in terrestrial ecosystems^25^. They also serve as a food source for birds and bats and are a vital link between plants and higher trophic level organisms in the food chain. Owing to their diversity, easy sampling with light traps, and close habitat associations, moths are frequently used for assessing species diversity against changes in landscape management^25,26^ and climate change^27,28^. For example, in many regions of the world, a marked decline in insects (including butterflies and moths) has been reported due to environmental changes, including climate change^27,29–32^.

In this study, we examined the diversity pattern of moth assemblages over the 12 years (2009–2020) at 11 study plots on an island mountain in HNP. The study was designed to address the following questions regarding the alpha and beta diversity of moths on an island mountain: How diverse are the moths on the island mountain? Is there any particular distribution pattern of moth species and individuals along the elevation and which environmental variables have caused the distribution of moth assemblages on the mountain? Have the diversity and composition of moth assemblages changed at this time?

## Results

### Alpha diversity of moths in Mount. Hallasan

The 28,507 specimens collected from 11 study plots represent 766 species in 14 families (Table 1, Supporting material Table S1). The estimated species richness, as calculated by the Chao 1 estimator, was 944.1 (lower and upper 95% confidence intervals, 886.5–1029.4). Across the 11 survey sites, a geometrid moth, *Alcis angulifera* was the most abundant, followed by the erebid moths, *Hydriollodes morosa,* and *Ghoria gigantean*.

**Table 1:** Information of the geographic location, elevation (m), vegetation type, sampling period, number of samplings, the number of individuals (abundance), and species (species richness, observed and Chao 1 estimated with lower and upper 95% confidence interval) of moths collected from 2009 to 2020 in Mount.

Site name	Geographic location			Vegetation type	Sampling period	TS	Diversity		
	Elevation (m)	Latitude	Longitude				Abundance	Species richness (observed)	Estimated species richness (Chao 1)
HRRL	278	33°18′57.0″	126°37′09.9″	EG	2009–2020	67	1799	306	453.8 (398.7, 541.6)
HRRH	525	33°19′56.7″	126°36′25.7″	EG	2009–2020	67	2632	321	437 (393.2, 507.4)
SPAL	645	33°23′06.7″	126°37′16.0″	TD	2009–2020	66	5373	330	531.3 (451.5, 663.4)
CWS	673	33°24′36.1″	126°29′43.3″	TD	2009–2020	67	3405	404	561.7 (506.1, 647.8)
SPAH	752	33°22′14.0″	126°37′31.6″	TD	2009–2020	68	3146	302	410.9 (367.0, 484.4)
YS	963	33°19′57.6″	126°27′52.6″	TD	2009–2020	66	4726	270	342.7 (311.4, 397.9)
ERM	954	33°23′31.6″	126°29′13.0″	TD	2009–2020	68	3492	327	433.0 (391.6, 501.0)
1100 T	1109	33°21′32.1″	126°27′44.4″	TD	2009–2020	65	2342	251	388.1 (331.5, 484.6)
SJB	1410	33°22′32.2″	126°29′58.8″	DC	2010, 2011, 2013–2020	47	965	157	256.6 (210.8, 341.5)
YSH	1630	33°21′31.3″	126°30′29.1″	Cf	2014–2020	30	329	69	123.3 (90.9, 203.7)
USOR	1699	33°21′43.5″	126°31′10.0″	Cf	2010–2020	35	298	61	156.8 (94.2, 337.4)
							28,507	766	944.1 (886.5, 1029.4)

Hallasan National Park (HNP), South Korea.

Vegetation: EG. Evergreen, TD, Temperate deciduous, DC, Mixed deciduous and coniferous, Cf, Coniferous. TS (Total sampling counts) differed because of the survey period and sampling failure due to harsh weather conditions or mechanical problems.

**Figure 1:**
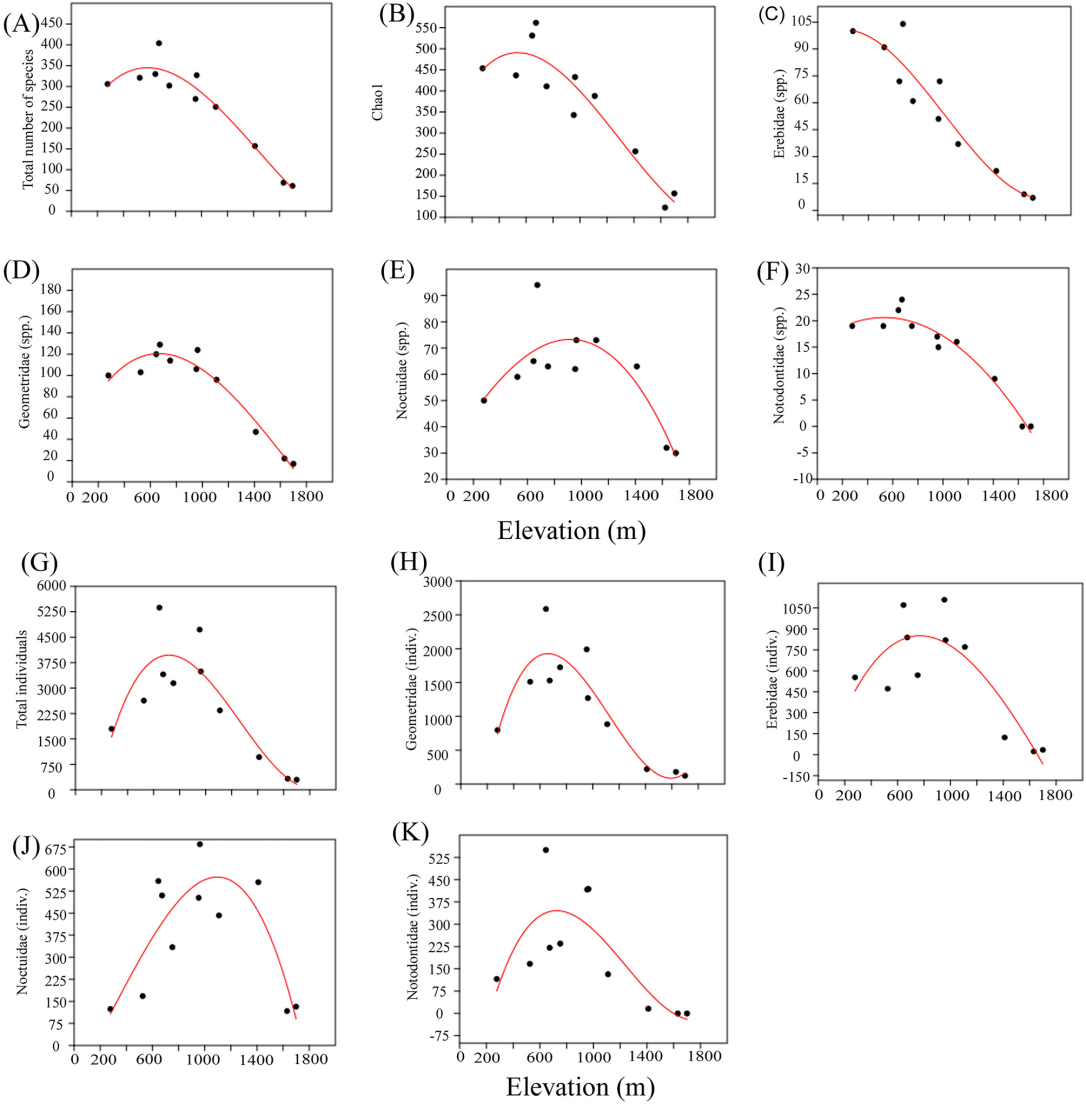
The best-fitted regression model for the species richness and abundance of moths along elevation in HNP. (**A**) Observed species richness, (**B**) Estimated species richness (Chao 1), (**C**) Species richness of the Erebidae, (**D**) Species richness of the Geometridae, (**E**) Species richness of the Noctuidae, (**F**) Species richness of the Notodontidae, (**G**) Total abundance, (**H**) Abundance of the Geometridae, (**I**) Abundance of the Erebidae, (**J**) Abundance of the Noctuidae, (**K**) Abundance of the Notodontidae.

In this study, we found that the number of moth species on HNP exhibited a hump-shaped pattern concerning elevation, from 280 to 1700 m, with a peak richness value at approximately 600–800 m (Fig. 1). Most families such as Geometridae, Notodontidae, and Noctuidae exhibited the hump-shaped pattern, whereas there was a monotonic decrease of the Erebidae with elevation. Similarly, the number of moth individuals on HNP exhibited a hump-shaped pattern with the peak occurring at 600–800 m, except for the species-rich family Noctuidae, which showed a peak at approximately 1000 m.

### Effects of environmental variables

Six environmental variables were tested for their relation to the number of observed species and the number of individuals on the island mountain. The number of individuals was affected by area and actual evapotranspiration (Akaike information criterion (AIC) = 189.76, Table 2). The number of observed species was affected by actual evapotranspiration (AIC = 126.08, Table 2).

**Table 2:** Summary of negative binomial GLM (generalized linear model) model to the number of individuals and the number of species from 11 study sites at HNP.

Independent variable	AIC	Z-value (Model parameter and standard error)			
		Intercept	Area	Plant species	AET
Number species	126.08	2.96** (1.58 ± 0.53)	− 1.42 (− 0.002 ± 0.001)	0.68 (0.01 ± 0.01)	5.57*** (0.01 ± 0.001)
Number individuals	189.760	1.27 (1.34 ± 1.06)	− 2.90** (− 0.01 ± 0.003)	− 0.29 (− 0.01 ± 0.02)	4.89*** (0.01 ± 0.002)

Tree dbh is the sum of dbh of trees in 20 × 20 quadrat.

*AIC* Akaike Information Criterion, *AET* actual evapotranspiration.

***P < 0.001.

**P < 0.01.

### Temporal changes

**Figure 2:**
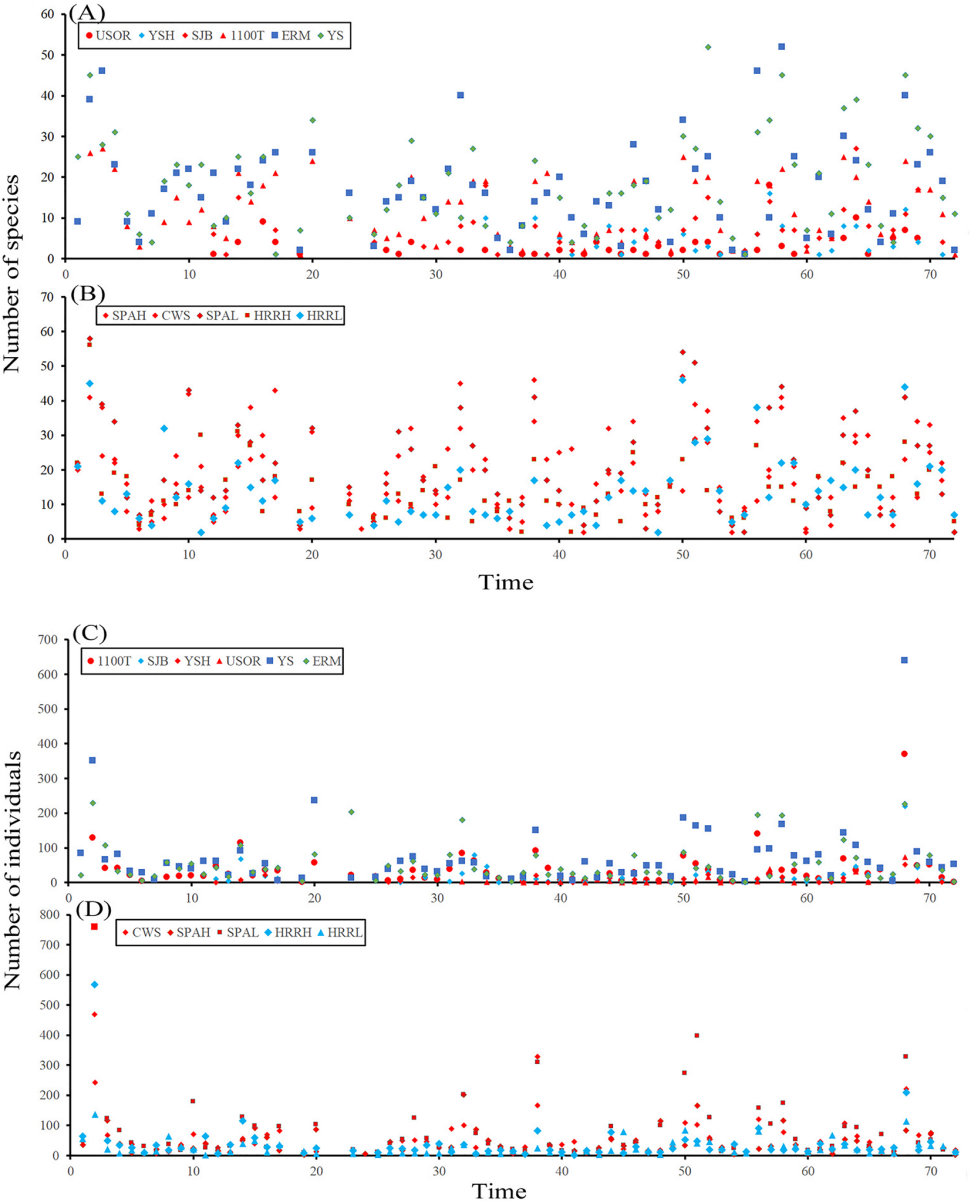
Scatter plot of the temporal changes in the number of species (**A**, **B**) and the number of individuals (**C**, **D**) of moths over the 12-year sampling at 11 study sites in HNP. The number of species (**A**) and individuals (**C**) at the sites is greater than 900 m a.s.l. The number of species (**B**) and individuals (**D**) at the sites is lower than 900 m a.s.l.

During the last 12 years, the species richness across most sites did not show any significant trend (Fig. 2, Table 3). No significant trend was also observed from 999 bootstrap resamplings (Table S3). However, the number of species of one of the highest elevation sites, SJB significantly increased (SJB: *t* = 2.21, df = 44, *P* < 0.05). Similarly, the number of individuals of most study sites did not show any trend, the exception being SJB and HRRH, which both showed a marginally decreasing trend (SJB: *t* = − 1.77, df = 44, *P* = 0.08, HRRH, *t* = − 1.69, df = 65, *P* = 0.09). The total assemblage of moths across the 11 sites showed an increasing trend in the number of species and a decreasing trend in the number of individuals but these trends were not significant (Fig. 2).

**Table 3:** Temporal changes in the number of species (species richness) and the number of individuals (abundances) of moths over the last 12 years in HNP. The results from a partial correlation trend analysis using the Pearson coefficient and the partial correlation coefficient are tested for significance with the student *t* distribution on df = n−2 degree of freedom.

Site	Number of species (covariate, number of individuals)			Number of individuals (covariate, number of species)		
	*t*	df	*P*-value	*t*	df	*P*-value
HRRL	1.48	65	0.14	− 0.78	65	0.44
HRRH	1.56	65	0.12	− 1.69	65	0.09
SPAL	− 0.34	64	0.73	0.06	64	0.95
CWS	1.47	65	0.15	− 1.36	65	0.18
SPAH	0.46	66	0.64	− 0.65	66	0.52
YS	− 0.14	64	0.89	0.65	64	0.52
ERM	0.76	66	0.45	− 0.92	66	0.36
1100T	− 1.04	63	0.3	0.78	63	0.44
SJB	2.21	44	0.03*	− 1.77	44	0.08
YSH	0.09	28	0.93	0.02	28	0.98
USOR	0.87	32	0.39	− 0.21	32	0.84
All combined	1.41	67	0.16	− 1.25	67	0.22

*P < 0.05.

### Beta diversity of moth assemblages

The highest dissimilarity of moth assemblages at 11 study sites was observed in the high-elevation zone, USOR (0.9 ± 0.004 s. d.), SJB (0.88 ± 0 s. d.), and the low-elevation zone, HRRL (0.84 ± 0.005 s. d.) and HRRH (0.85 ± 0.003 s. d.) (Table 4).

**Table 4:** Results from the partitioning of beta diversity of moth assemblages at 11 sites in HNP.

	HRRL	HRRH	SPAL	CWS	SPAH	YS	ERM	1100 T	SJB	YSH	USOR
SumS	779	794	1050	1141	865	899	952	650	295	127	107
Tsp	306	321	330	404	302	270	327	251	157	69	61
Shared_sp_	473	473	720	737	563	629	625	399	138	58	46
β_SIM_ (s.d.)	0.78 (0.006)	0.81 (0.007)	0.74 (0.005)	0.79 (0.005)	0.78 (0.009)	0.73 (0.005)	0.77 (0.007)	0.81 (0.004)	0.83 (0)	0.73 (0.005)	0.81 (0.007)
β_NES__(_s.d.)	0.07 (0.007)	0.04 (0.005)	0.05 (0.006)	0.04 (0.008)	0.04 (0.006)	0.06 (0.003)	0.04 (0.005)	0.03 (0.003)	0.05 (0)	0.06 (0.003)	0.09 (0.008)
β_SOR__(_s.d)	0.84 (0.005)	0.85 (0.003)	0.79 (0.004)	0.83 (0.005)	0.82 (0.004)	0.79 (0.004)	0.82 (0.005)	0.84 (0.005)	0.88 (0)	0.79 (0.004)	0.9 (0.004)

Sum S, the sum of the species richness values of all years; T_sp_ the total richness in the dataset; Shared_sp_, the number of shared species; β_sor__=_ β_SIM__+_ β_NES_, β_sor,_ Sørensen pairwise dissimilarity; β_SIM,_ the Simpson-based multiple-year dissimilarity (temporal turnover); β_NES,_ the nestedness component of the beta diversity; beta diversity data were obtained from simulations of 100 times across 10 years.

The total dissimilarity was relatively higher in the highest elevation site (USOR, βsor = 0.90), including a forest fire site (SJB, βsor = 0.88) (Table 4). The low-elevation sites, HRRH and HRRL also showed relatively higher total dissimilarity (HRRH, βsor = 0.85, HRRL βsor = 0.84). Despite the relatively higher dissimilarity, the species composition during the last 12 years was largely affected by active temporal turnover (SJB, βsim = 0.83, USOR βsim = 0.81, HRRH βsim = 0.81) but the effect of species loss was small (SJB, βsne = 0.05; HRRH, βsne = 0.04, USOR, βsne = 0.09).

**Figure 3:**
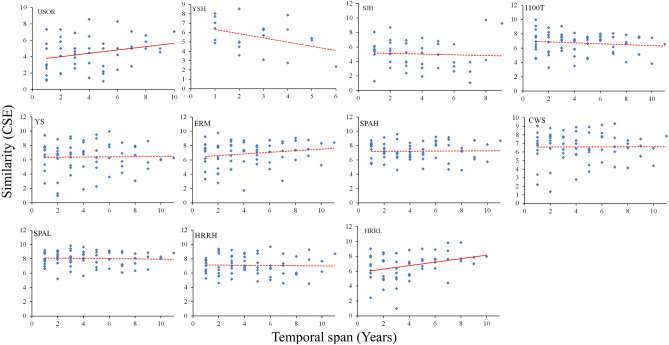
The similarity of moth assemblages at 11 sites against the temporal span from 2009 to 2020 in HNP. The similarity index was calculated by Chao-Sørensen estimated abundance-based (CSE) index. The solid line indicates the significant relationship between the similarity and time span at *P* < 0.05.

The composition of moth assemblages during the last 12 years showed no significant trend except for two sites, HRRL and USOR (Fig. 3). At most sites the moth assemblages had a similar composition. However, the uppermost and lowermost sites showed that the moth assemblages had significant changes in similarity over a relatively long period.

## Discussion

### Pattern and mechanism of the diversity of moths on Mount Hallasan

The data presented in this study were collected over 12 years from different elevations in HNP. We employed systematic and constant sampling with automatic UV light traps, which were run simultaneously and for the same number of hours at all sampling sites. The pattern of diversity in this study reflects the flight activity of light-attractant adult moths^33^. There were a few missing data points, mainly due to harsh weather conditions or mechanical failure (Table 1). Nevertheless, we conclude that the analyses and the results are robust because the data were obtained over 12 years: the sampling data can be considered as a proxy for absolute abundance^33^.

The numbers of moth species and individuals in HNP exhibited a hump-shaped pattern with a peak around 600–800 m (Fig. 1). This hump-shaped pattern of diversity matches one of the dominant patterns along the elevational transects^20,34,35^. In the case of two families, however, there were notable exceptions to the general trend patterns in elevation-diversity studies (Fig. 1): the Erebidae exhibited a monotonic decrease in the number of species and the Noctuidae showed the highest peak of the number of species and individuals at approximately 1000 m. The moth families present various ecological adaptations, including thermal strategies^36^. Thermoregulation in insects is a well-known phenomenon using behavior (e.g., control of solar input posturally by basking in the sun or raising their bodies away from the hot ground) and physiological mechanism (e.g. shivering by simultaneous stimuli of flight muscles and preventing overheating by increased heartbeat rate)^37,38^. Elevational clines in body size and color lightness have often been observed in moth families^39^: noctuid moths show darker coloration and larger body size in colder environments, whereas geometrid moths show no relationship between coloration and body size. Instead, the number of species of Larentiinae, one of the subfamilies of the Geometridae, increases as elevation increases^25,34,40,41^. They often show remarkable physiology that allows for flight activity during winter time^42^ and survival strategies by flying in the predator-free environment and by feeding herbs, where insectivorous bats and woody hostplants are rare^34,43,44^. In this study, this pattern was partly congruent with relatively higher numbers of species and individuals of Noctuidae along the high elevation in HNP.

In addition to noctuid moths, large-bodied moths, such as species of Sphingidae and Saturniidae, need to raise body temperatures above the ambient temperature to take flight^36^. The pre-flight warm-up together with the high wing load in Noctuidae, Sphingidae, and Saturniidae is very energy-consuming. Thus, the large body size reduces the loss of body temperature, which is beneficial to these moths in colder environment^39^, which might explain the fact that the peak of species richness of these moth families is at higher elevations. We found that in HNP, the highest species richness of Sphingidae and Saturniidae was recorded at one of the high-elevation sites, YS (963 m a.s.l.) (Fig. S2), compared, for example, with Geometridae at SPAH (752 m a.s.l.).

One of the most species-rich families of Lepidoptera, the Erebidae, is comprised of several well-known subfamilies, including Herminiinae, Arctiinae, and Lymantriinae^45^. It has been noted that the species richness of Arctiinae decreases with increasing elevation in tropical mountains^46^. Although plant diversity is known to be an important factor affecting moth diversity because moths generally have herbivorous larvae, which tend to be specialized in host plant use^12,33,46^, the abundant resources in high elevation (e.g. algae and lichens for Ctenuchini arctiines) do not harbor many moth species but few species adapted to the upper cloud forest zone^33,46^. Similarly, in our study in HNP, the number of species of the Erebidae (including Arctiinae) decreased with increasing elevation and the common species in high elevation included the lichen-feeder (*Collita griseola*) or dead leaves-feeder (*Hydrillodes morosa*). We conclude that, in the case of Erebidae, the decreasing pattern of species richness along a gradient of elevation is consistent in temperate and tropical mountains but a few species dominate the harsh condition.

The best-known relationship between area and the number of species is often explained by the availability of habitat and resources since larger areas that comprised diverse habitats and ample resources support a large number of species in a given area^8^. In most mountain regions, the area declines monotonically with elevation but the species richness largely peaks at mid-elevation^18,20,34,35^. Many hypotheses were tried to explain this pattern including geometric constraints, phylogenetic history, past climatic variation, turnover at ecotones, specific biotic interactions, area-integrated productivity, and anthropogenic disturbance^13^. We found that the elevational area was not the driving variable for the number of species but the number of individuals. The negative relationship between the number of individuals and the elevational area could be attributed to a few dominant species in high elevations.

**Figure 4:**
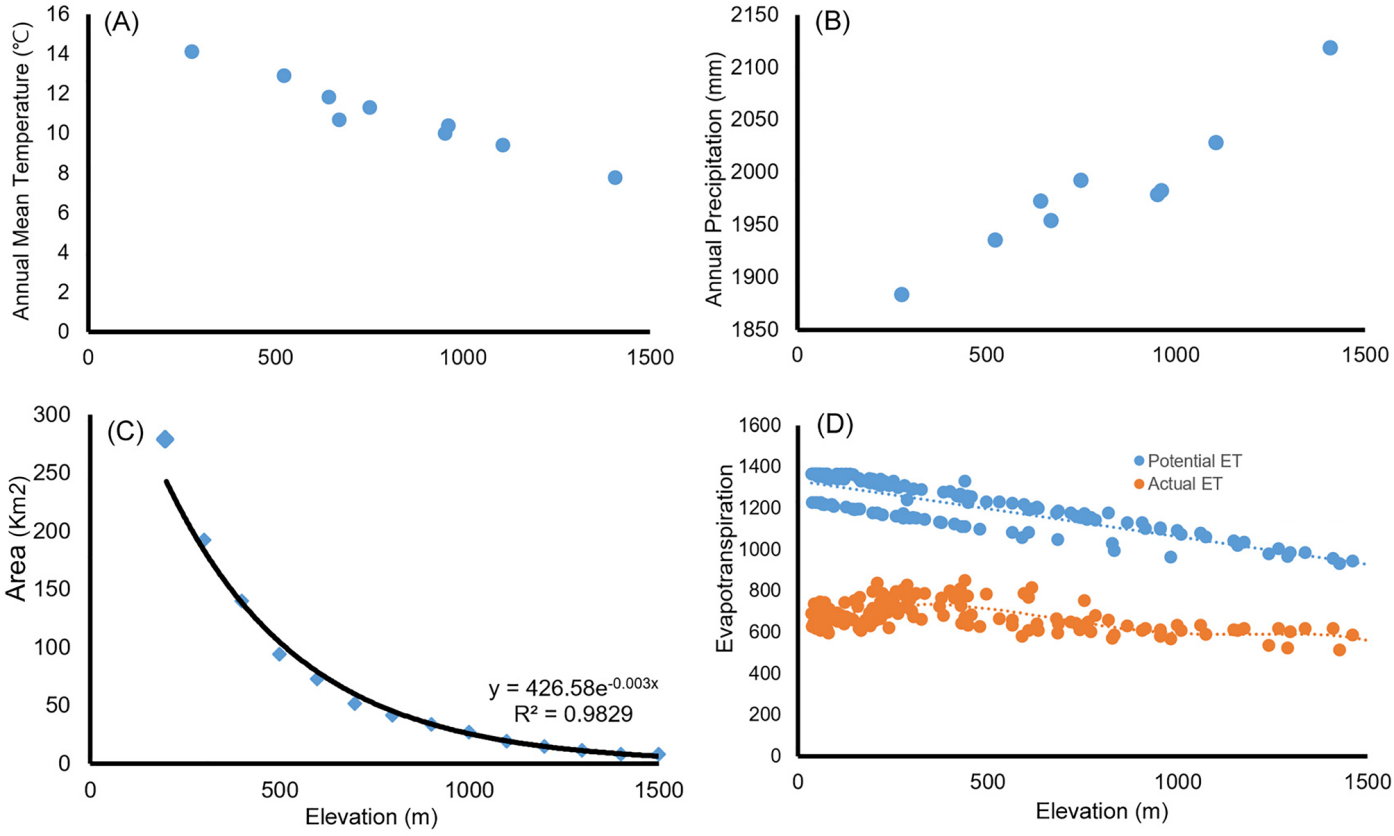
Changes in (**A**) annual mean temperature (°C), (**B**) annual precipitation (mm), (**C**), area (Km^2^), and (**D**) potential ET (evapotranspiration) and actual ET (evapotranspiration) (mm) along elevation in HNP.

The asymmetric rate of decline in diversity at mid-point elevations has been documented for many taxa (e.g., ants and geometrid moths) on different tropical mountains^47,48^. The higher loss of species diversity below the mid-point is probably due to deforestation or habitat disturbance in low-elevation areas^49^. The elevational pattern of the total diversity of moths in the present study differed somewhat from this trend, with species diversity being high at low elevations. This could be due to either the largely missing data from the lower-elevation areas or the result of the smaller land area of the island mountain (Fig. 4). The lower-elevation area of Jeju-do Island (300–400 m a.s.l.) is characterized by many low volcanic hills (“O-reum” in Korean), and these low volcanic hills are covered with coniferous and deciduous mixed vegetation at low and middle elevations and grasses on the hilltops. The habitat heterogeneity of this habitat and the massive postglacial colonization are the reason for the diversity of insects^50^ and genetic diversity of the plants in this area (e.g., *Quercus* species)^51^. However, high developmental pressure on the coastal and low-elevation areas of the island has resulted in a deterioration of this biodiversity^52^. Because most of our study sites in the northern aspect were above the boundary of the national park, the diversity pattern below 500 m was excluded. Additionally, the high-elevation areas of the island mountain in our study area are smaller, thus limiting the total number of moth species and individuals.

The plot of the number of individuals against elevation is hump-shaped and nearly symmetrical (Fig. 1). Although the number of species was small at high-elevation sites, there were a few dominant species of Geometridae (*Alcis angulifera, Menophra senilis, Parectropis nigrosparsa*), Noctuidae (*Diarsia pacifica*), and Erebidae (*Hydriollodes morosa, Ghoria gigantea, Dolgoma cribrata*) that occurred widely across all elevations (Fig. S1). This phenomenon, known as density compensation, explains the higher abundance of some species due to competition or predator relaxation^4–6^. The altitudinal distribution of *Alcis angulifera,* a polyphagous geometrid moth, is an example of density compensation: the species was found at all survey sites but was in higher abundance at low and mid-elevations (between 525 and 752 m). A noctuid moth *Diarsia pacifica*, feeding on composite plants (Asteraceae) occurred at all survey sites but was in higher abundance at high elevations (1410 m). The high number of occurrences of these common moth species on the island mountain could be the result of adaptive strategies, in particular, the broad range of food plants utilized by these species or the fact that there is less predation at higher elevations (e.g., Larentiinae geometrid moths, see above).

On the other hand, the diversity of moths was less affected by plant species richness (Table 2). The magnitude and direction of the relationship between tree species diversity and herbivores can be affected by plot size, tree age, and tree density^53^. The abundance of herbivores is related to host tree density: the resource concentration hypothesis^54^ postulates that herbivores especially, specialist are more abundant at higher host plant densities; but the resource dilution pattern^55^ shows that the herbivores are more abundant in low-density patches. We found that the dominance of a few moth species (e.g., *Alcis angulifera, Menophra senilis, Parectropis nigrosparsa, Diarsia pacifica*, *Hydriollodes morosa, Ghoria gigantea, Dolgoma cribrata*) in the current study areas show the resource concentration pattern. This could be explained by the persistence of moth populations in a forest. The forest ecosystem is spatially and temporally stable because the forest patches are not created every year, compared to the agricultural ecosystem. These stable conditions enable the herbivores to build up their populations on host tree patches consistently^53^. This suggests that the high abundance of tree species favors a few common species but disfavors the overall abundance of moth species.

Among environmental factors, the most significant variable affecting both the numbers of moth species and individuals was actual evapotranspiration (Table 2). The diversity of most species was strongly correlated with energy-dependent variables, such as temperature, precipitation, and actual evapotranspiration^56,57^. Actual evapotranspiration, deriving from temperature, precipitation, and estimated soil water storage^58^ acts as a strong predictor of global plant and animal species richness^57,59–61^. Temperature is one of the strongest ecological factors affecting the distribution of plants and animals, particularly ectotherms, such as insects^12,48,56^. Brehm et al*.*^12^ reported a negative relationship between humidity and the diversity of geometrid moths in Costa Rica. In contrast, Beck and Chey^48^ showed a weak but significant positive correlation between humidity and moth diversity in Borneo. The annual mean precipitation in HNP over the past decade ranged from 1884 to 2119 mm, which is wetter than the lowlands of the island. High rainfall increases larval and pupal mortality through fungal infection^62^. On the other hand, increased rainfall influences the growth of vegetation, a larval food source, that continuously supports moth populations^63^. Kreft and Jetz^64^ also indicated that water-related parameters and other predictor variables, such as habitat heterogeneity, played a significant role above a threshold of energy limitation. We suggest that the moth species diversity on the island mountain in our study is strongly correlated with an energy-related variable, namely, actual evapotranspiration. Potentially, changes in temperature and precipitation patterns brought on by climate change could have a strong impact on the species richness and abundance of moths on the island mountain.

### Temporal trends of moth diversity on Mount Hallasan

The overall moth populations have fluctuated nonlinearly over the past decade (Fig. 2), and the moth populations at most study sites showed no significant trend of change during these periods (Table 3). Species assemblages in a community can be transformed actively by species introduction, extirpation, or changes in the extent of their range. As suggested by Lennon et al.^65^, the highest α‐diversity areas produce higher similarity (lower β‐diversity) because there is less chance for differences in species composition when a higher proportion of the species pool is present. At both ends of the elevational gradient, however, there was a significant change in the composition of the moth assemblages: at the low-elevation site, HRRH, the number of individuals tend to be decreased (*t* = − 1.69, *P* < 0.1); and at the highest-elevation site, SJB, the number of species significantly increased (*t* = 2.21, *P* < 0.05) and the number of individuals tend to be decreased (*t* = − 1.77, *P* < 0.1, Table 3).

After a forest fire at SJB in 2012, the moth assemblage showed both a drastic change in species composition and quick recovery within 5 years^66^. This is consistent with what is generally known about the taxonomic composition of high-elevation sites; harsh weather often results in local extirpation of species and, consequently, highly variable community composition^20^. We observed that the total dissimilarity was relatively higher in the highest elevation site (USOR), including a forest fire site (SJB)(Table 4). In addition, the low-elevation sites, HRRH and HRRL showed relatively higher total dissimilarity. Despite the relatively higher dissimilarity, the species composition during the last 12 years was affected by active temporal turnover with a high βsim value between 0.73 and 0.83. It should be noted that the beta-diversity pattern in the highest site is the result of both species replacement across elevations and species loss towards the highest elevations, whereas the beta-diversity pattern in the low-elevation or recently disturbed site is mostly caused by species replacement. This combined effect of temporal turnover is possibly due to unexpected events, such as forest fires and recent warm weather conditions, which may increase species richness and the high turnover rate of moth assemblage in the high-elevation sites, including the SJB site.

Unlike grassland-inhabiting butterflies and moths, which have dramatically declined over recent decades^67^, woodland species have exhibited greater stability in terms of abundance and diversity, possibly due to the buffering effect (microclimate) of forest cover^68,69^. The relatively stable population of moths in HNP over recent decades could be because most species in the analysis are woodland species. On the other hand, this trend could be affected by future climate change as the species occurring in the southern part of England have declined more strongly than those in the northern part^70^. Because we focused on the elevational zones of a mountain, the trends in assemblage composition we observed are expected to differ somewhat from the reported cases of dramatic decline^31^. Over the past decade, moth assemblages across 11 study sites in HNP were not drastically changed in diversity and abundance; however, the lowest and highest elevation sites (HRRL, SJB) showed a significant change in species composition. Given the elevation and the location of the study area, the impact of climate change may vary according to the elevational zone, with greater impact at the lower- and higher-elevation zones and less of an impact at the middle-elevation zone.

Long-term ecological monitoring (more than 10 years) can help to identify complex ecological patterns and processes^71^. Currently, many insect species have shown a decline in diversity and abundance^30,72^. Sánchez-Bayo and Wyckhuys^32^ reviewed the status of the decline of moths and butterflies in Europe, North America, and Asia and estimated the global rate of decline to be approximately 40% over the next few decades. Didham et al*.*^73^ listed several critical challenges in drawing robust conclusions about the decline in the insect population. Among them, the establishment of a historical baseline is essential to estimate the actual historical trends. Similarly, Sánchez-Bayo and Wyckhuys^32^ argued that the extent of the losses was difficult to quantify due mainly to the lack of historical knowledge in many regions, and many subtropical, and tropical countries. On the Korean peninsula, there is a lack of survey data (in the case of moths) before 2000. Since 2005, long-term monitoring data has been collected through the Korea Long-Term Monitoring Project (KLTER). The comparison of the estimated species richness from the last 12-years data shows no drastic decline in the species richness of moths in HNP. Notwithstanding any significant trend in the species richness of moths on the island mountain, the long-term monitoring of moth assemblages along elevational transects could provide important insight into the effects of climate change on moth populations.

## Materials and methods

### Study area

**Figure 5:**
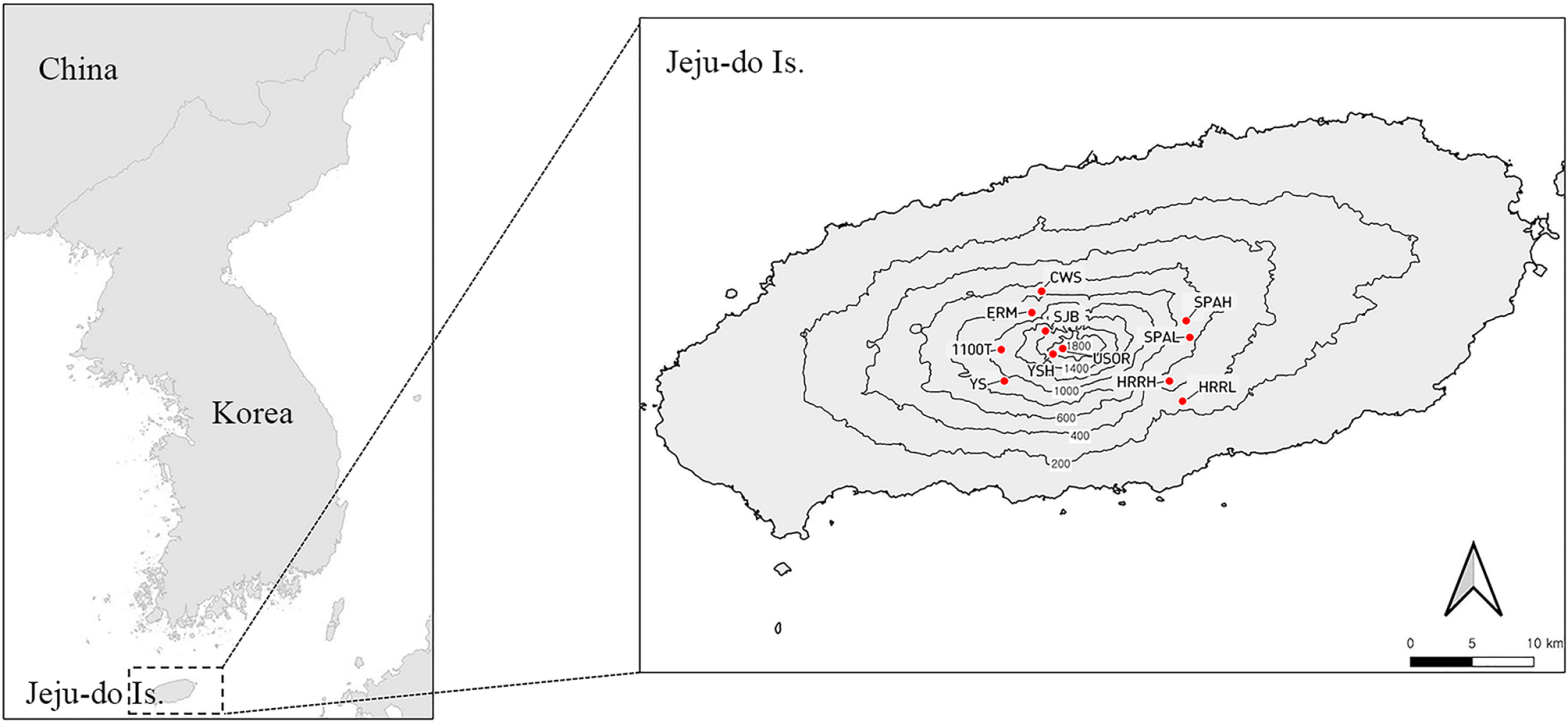
Map of 11 study sites in Mount. Hallasan National Park (HNP), South Korea.

Approximately 3240 islands are distributed off the coast of South Korea, and more than 99% of these islands are continental; only a few, such as Jeju-do and Ulleung-do, are volcanic. The 149 km^2^ Mount Hallasan National Park (HNP), named after South Korea’s highest peak, Mount Hallasan (1950 m above sea level) is located on South Korea’s largest volcanic island, Jeju-do (126° 09′ 42″–126° 56′57″ E, 33° 11′ 27″–33° 33′ 50″ N, with an area of 1833.2 km^2^) (Fig. 5). The elevational gradient of HNP is characterized by vertical stratification of vegetation zones, from boreal to evergreen deciduous, producing a unique pattern of alpine vegetation and high species richness, quite unlike the environment of most of the lower-elevation in South Korea^24^. The climate of Jeju-do Island is generally regarded as temperate, although the coastal and lower elevation areas are warm-temperate. Recently, the climate of the island has grown wetter and hotter; the mean temperature has increased by approximately 1.4 °C from 1987 to 2017, and precipitation has increased by 124 mm^74^.

### Sampling and data

The 11 survey study plots for collecting moths ranged from the evergreen deciduous tree zone (HRRL) to the subalpine tree zone (SJB) on HNP (Table 1, Fig. 5). The moth populations were sampled once a month, from May to October, from 2009 to 2020. A 22-W ultraviolet circline light tube with a 12 V battery (BioQuip Co., USA) was used to collect moths at each study plot. Moth sampling was conducted for only 5 h after dusk due to the limited battery power. The light traps were operated simultaneously at all 11 sites to minimize sampling bias. The moths were identified at the species level by the first author (SWC). Moth identification referred to the taxonomic literature^75–78^.

A 6-year dataset (2013–2018) was published and is available online (https://doi.org/10.3897/BDJ.8.e51490). The complete dataset from 2009 to 2020 is provided in the supplementary material (Table S1). Due to the forest fire at the SJB site in April of 2012, sampling at the higher-elevation sites was stopped at this time, and we only sampled three times at lower-elevation sites. At other times, the cold weather or light trap malfunction caused the failure of sampling and we treated this as missing data.

### Environment

The data for temperature and precipitation between 1970 and 2000 were extracted from WorldClim datasets [ver. 2, spatial resolution 30 s (~ 1 km^2^), Fig. 4]. The actual and potential evapotranspiration data were acquired by applying the transformed model of the Soil and Water Assessment Tool from USDA-ARS to four watersheds on Jeju-do Island (elevation ranges 36–1576 m, n = 144; SWAT-K model)^79^. Because both actual and potential evapotranspiration were highly correlated (Pearson *r* = 0.91, *P* < 0.001), we utilized only the actual evapotranspiration in this analysis. The land area of the 100-m elevational bands of Jeju-do Island was calculated from raster map data available from Opentopography (https//opentopography.org) using Q-GIS (ver. 2.18.10.v).

Because the environmental data might deviate from the values at actual sites, fitted regressions of the elevation and the environmental variables were used^80^ based on polynomial regression analysis with the lowest Akaike information criterion (AIC, lower AIC value indicates a better fit of the model) and highest R^2^ (area y = 519.2 − 1.56x + 0.002x^2^ − 1.05E − 06x^3^ + 2.17E − 10x^4^, AIC = 125.36, R^2^ = 0.99; actual evapotranspiration y = 614.5 + 0.74x − 0.001x^2^ − 3.63E − 07x^3^ + 1.21E − 09x^4^ − 4.57E − 13x^5^, AIC = 2.89 × 10^5^, R^2^ = 0.48).

Plant species richness, number of trees, and the total diameter of trees of each study site were obtained from 20 × 20 m quadrats in which all trees with diameters at breast height (dbh) greater than 2 cm were counted, and shrubs and herbs were counted in four 5 × 5 m subplots within each quadrat.

### Data analysis

The number of species (species richness) and the number of individuals (abundance) were obtained at each study site, and the estimated number of species (Chao 1 based on abundance) was calculated. Chao 1 is a robust estimator of minimum richness and performed better estimation in terms of bias, precision, and accuracy^81^.

Polynomial regression analyses were performed to determine the distribution patterns (linear or hump and sigmoid‐shaped) of the numbers of observed and estimated (Chao 1) species and the number of individuals of the total and the four species-rich families of Lepidoptera (Geometridae, Erebidae, Noctuidae, and Notodontidae) at a particular elevation. The best polynomial regression was selected by comparing the R^2^ and the corrected AIC value of first, second, and third‐order polynomial regressions, and was calculated using PAST software version 4.11 (http://www.nhm.uio.no/)^82,83^.

The relationship between the numbers of moth species and individuals in HNP and the six environmental variables (elevation, area, plant species richness, number of tree individuals, total tree dbh, and actual evapotranspiration) was analyzed. Before the analysis, we checked the multi-collinearity by calculating a variance inflation factor (VIF) for each variable using the VIF function from the ‘car’ package. The VIF is an estimate of the proportion of variance in one predictor explained by all the other predictors in the model^80^. We removed three variables with the highest VIF (threshold < 3) (elevation, number of tree individuals, and total tree dbh) and analyzed using the negative binomial GLM (generalized linear model) than a Poisson GLM because the data set in the current study showed an overdispersion.

The number of species (species richness) and the number of individuals (abundance) of each study site over the last 12 years were used to detect a partial correlation trend test using the Pearson correlation coefficient in “partial.cor.trend.test” of the “trend” package in R software version 4.0.3 (http://www.r-project.org/)^84^. The null hypothesis was that the partial correlation coefficient is zero: the number of species that are correlated with time showed no trend when the effect of co-variate, the number of individuals was partialled out, and the number of individuals that are correlated with time showed no trend when the effect of co-variate, the number of species was partialled out. The alternative hypothesis was that the number of species or the number of individuals showed a trend^85,86^. In addition, we resampled the data 999 times for a bootstrap test because the number of sampling at each study site was different mainly due to weather conditions or mechanical failure. The bootstrap test was done using the “trend.test” of the “pastecs” package in R software version 4.0.3 (http://www.r-project.org/) ^84^.

The beta diversity of moth assemblages expressed as the similarity among the sampled years was calculated using Chao-Sørensen estimated abundance-based (CSE) index. The CSE is a non-parametric index that does not assume any particular species abundance distribution to derive the estimators. The similarity index is corrected for unseen species and is less affected by under-sampling bias^87^. The similarity matrix for each study site was obtained by calculating the degree of shared species of moth assemblages consecutively among the sampled years. These indices of the estimated species richness and similarity were obtained using the software, EstimateS version 9.1 (http://www.robertkcolwell.org/pages/estimates/)^88^, and the linear model of the similarity against year interval for the study sites was performed using R software version 4.0.3 (http://www.r-project.org/) ^84^.

Beta diversity can be partitioned into two components^89^: spatial turnover and nestedness. Spatial turnover is the replacement of some species with others in neighboring communities, through environmental sorting or spatial and historical constraints^89^. In contrast, nestedness is a measure of species loss in which an assemblage is a subset of another, reflecting a non-random process of species loss due to the orderly disaggregation of assemblages^89^. Baselga^89^ proposed the method to partition total beta diversity into two additive components accounting for spatial turnover and nestedness: β_sor_ = β_SIM_ + β_NES_, where β_sor_ is Sørensen pairwise dissimilarity, β_SIM_ is the Simpson-based multiple-site dissimilarity (spatial turnover), and β_NES_ is the nestedness component of the beta diversity. We calculated these indices for each site and four elevation zones using the “betapart” package in R software version 4.0.3 (http://www.r-project.org/) ^84^.

We compared the three measures of dissimilarities (turnover, nestedness-resultant fraction, and overall beta diversity) after resampling 100 times for a subset of two study sites in each elevation zone. Similarly, we resampled 100 times for a subset of 10 years in each study site. We used the Sørensen index for calculating dissimilarity and the values from resampling were compared by ANOVA. To calculate multiple site dissimilarity, the function, beta.sample in “betapart” package was employed^89,90^.

## Data availablity

All data generated or analyzed during this study are included in this published article and its supplementary information file.

## Supplementary Information

Supplementary Information 1.

Supplementary Information 2.

Supplementary Information 3.

Supplementary Information 4.

Supplementary Information 5.
