# Supplementary figures and images for: Spatial and temporal changes in moth assemblages along an altitudinal gradient, Jeju-do island
Source: Sci Rep. 2022 Nov 29;12:20534. doi: 10.1038/s41598-022-24600-z (PMC9709042; doi:10.1038/s41598-022-24600-z)

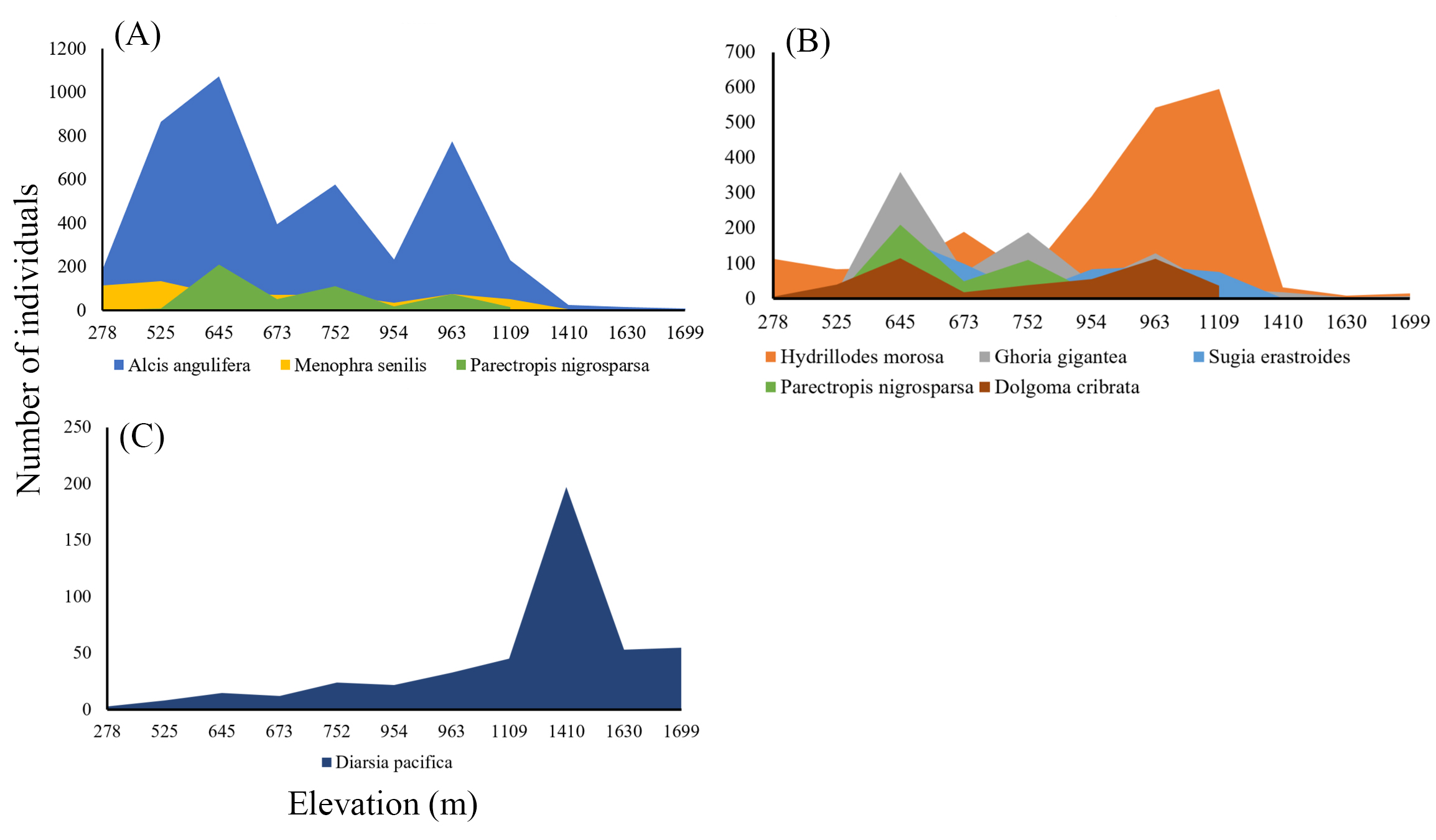

Supplement: Supplementary file 1 — Supplementary Information 1. [file 41598_2022_24600_MOESM1_ESM.jpg]

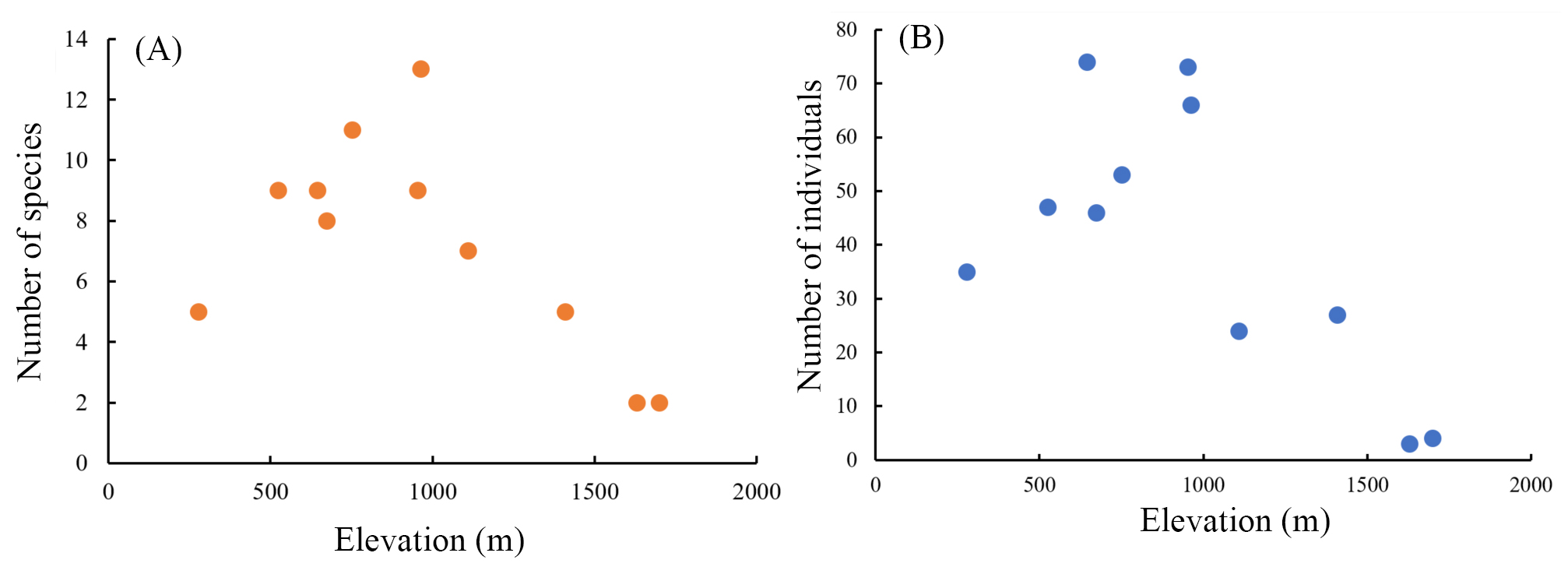

Supplement: Supplementary file 2 — Supplementary Information 2. [file 41598_2022_24600_MOESM2_ESM.jpg]
