# Supplementary material for: Spatial and temporal changes in moth assemblages along an altitudinal gradient, Jeju-do island
Source: Sci Rep. 2022 Nov 29;12:20534. doi: 10.1038/s41598-022-24600-z (PMC9709042; doi:10.1038/s41598-022-24600-z)
Supplement: Supplementary file 4 — Supplementary Information 4. [file 41598_2022_24600_MOESM4_ESM.docx]

Table S2. Polynomial regression for the species richness and abundance of moths along elevational gradient in HNP (richness as a function of elevation, elevation^2^ and elevation^3^).

|  | Number of species | Order 1 | | Order 2 | | Order 3 | | |  |
| --- | --- | --- | --- | --- | --- | --- | --- | --- | --- |
|  |  | R^2^ | AIC | R^2^ | AIC | | R^2^ | AIC | |
| Total | 766 | 0.78 | 26822 | 0.93 | 8713.3 | | **0.94** | **7516.3** | |
| Chao 1 | 944.1 | 0.78 | 44396 | 0.87 | 25836 | | **0.88** | **23177** | |
| Geometridae | 248 | 0.70 | 4913.8 | 0.95 | 824.92 | | **0.96** | **718.43** | |
| Erebidae | 187 | 0.89 | 1297 | 0.89 | 1297.1 | | **0.90** | **1182.3** | |
| Noctuidae | 201 | 0.26 | 2475.5 | 0.73 | 920.98 | | **0.73** | **919.44** | |
| Notodontidae | 36 | 0.81 | 129.39.5 | **0.96** | **41.04** | | 0.96 | 44.60 | |
|  | **Number of individuals** | **Order 1** | | **Order 2** | | | **Order 3** | | |
|  |  | R^2^ | AIC | R^2^ | AIC | | R^2^ | AIC | |
| Total | 28,507 | 0.38 | 1.73*10^7^ | 0.73 | 7.54*10^6^ | | **0.79** | **5.84*10^6^** | |
| Geometridae | 12,821 | 0.45 | 3.53*10^6^ | 0.69 | 2.01*10^6^ | | **0.84** | **1.03*10^6^** | |
| Erebidae | 6,384 | 0.39 | 9.17*10^5^ | 0.73 | 4.06*10^5^ | | **0.74** | **3.99*10^5^** | |
| Noctuidae | 4,127 | 0.01 | 4.30*10^5^ | 0.68 | 1.37*10^5^ | | **0.71** | **1.28*10^5^** | |
| Notodontidae | 2,273 | 0.25 | 2.58*10^5^ | 0.55 | 1.55*10^5^ | | **0.62** | **1.33*10^5^** | |

Bold numbers indicate the best regression model selected by lowest AIC value.
