# Supplementary material for: Spatial and temporal changes in moth assemblages along an altitudinal gradient, Jeju-do island
Source: Sci Rep. 2022 Nov 29;12:20534. doi: 10.1038/s41598-022-24600-z (PMC9709042; doi:10.1038/s41598-022-24600-z)
Supplement: Supplementary file 5 — Supplementary Information 5. [file 41598_2022_24600_MOESM5_ESM.docx]

| Sites | Number of species | | | | Number of individuals | | |  |
| --- | --- | --- | --- | --- | --- | --- | --- | --- |
|  | ρ (rho) | bias | SE | P-value | ρ (rho) | bias | SE | P-value |
| SPAH | -0.04 | 0.04 | 0.13 | 0.38 | -0.02 | 0.02 | 0.12 | 0.43 |
| SPAL | -0.10 | 0.10 | 0.12 | 0.21 | -0.06 | 0.06 | 0.13 | 0.31 |
| ERM | -0.02 | 0.03 | 0.12 | 0.44 | -0.03 | 0.03 | 0.12 | 0.39 |
| YS | 0.06 | -0.06 | 0.12 | 0.67 | 0.13 | -0.12 | 0.12 | 0.82 |
| CWS | 0.02 | -0.01 | 0.12 | 0.56 | 0.03 | -0.03 | 0.12 | 0.61 |
| HRRH | 0.00 | 0.01 | 0.13 | 0.47 | -0.06 | 0.07 | 0.12 | 0.28 |
| HRRL | 0.17 | -0.16 | 0.12 | 0.91 | 0.17 | -0.18 | 0.12 | 0.93 |
| SJB | 0.20 | -0.20 | 0.15 | 0.09 | 0.08 | -0.07 | 0.15 | 0.28 |
| 1100T | -0.09 | 0.09 | 0.13 | 0.24 | -0.03 | 0.03 | 0.13 | 0.39 |
| USOR | 0.27 | -0.27 | 0.18 | 0.07 | 0.23 | -0.23 | 0.17 | 0.12 |

Table S3. Summary of a bootstrap test for the number of species and individuals from 11 sites of HNP. 999 times are resampled for a bootstrap test between the observation and the time using a non-parametric Spearman test to check whether the series has an increasing or decreasing trend. SE. standard error.
